# Supplementary material for: Malaria oocysts require circumsporozoite protein to evade mosquito immunity
Source: Nat Commun. 2022 Jun 9;13:3208. doi: 10.1038/s41467-022-30988-z (PMC9184642; doi:10.1038/s41467-022-30988-z)

Supplementary information

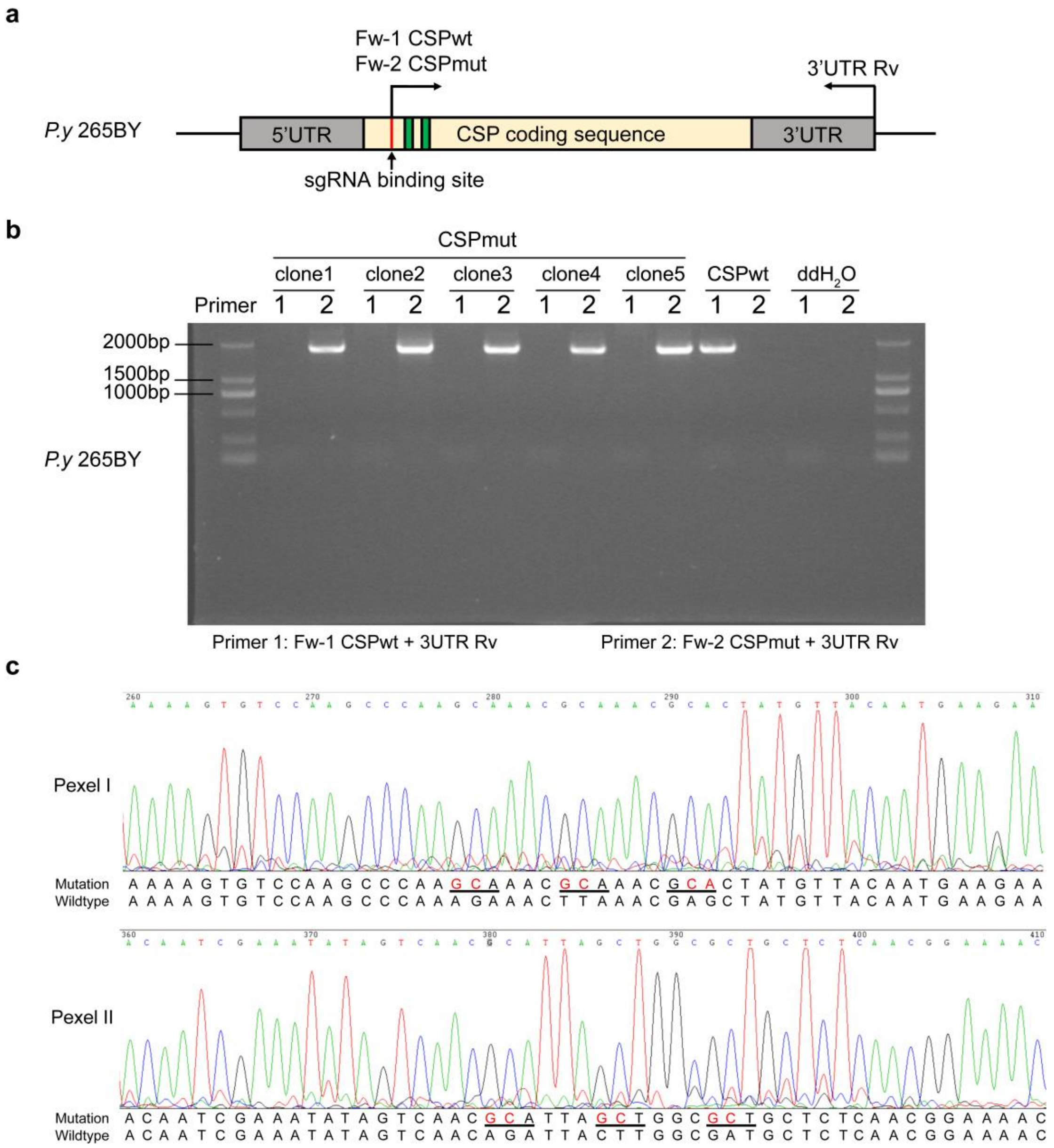

Supplementary Figure 1. Identification of *P. yoelii* CSP$_{mut}$ parasite clones.

a Primers designed to identify WT and mutant parasites. **b** ~2.0 kb fragment was amplified from the genomic DNA extracted from mutant parasites, with Primer 2 (Fw1-2 (Mut) and 3′-UTR Rv). No fragment was obtained with Primer 1 (Fw1-1 (WT) and 3′-UTR Rv). **c** The pyrimethamine-resistant parasites were collected and cloned by injecting each mouse with 100 µL of PBS-diluted parasite solution containing ~1.0 infected RBC. The resulting *P. yoelii* CSP$_{mut}$ parasite clones were identified by PCR. **c** The *CS* gene of *P. yoelii* CSP$_{mut}$ parasites were amplified by PCR and sequenced. The underlined nucleic acid sequence indicates the successful pexel I/II domain mutation.

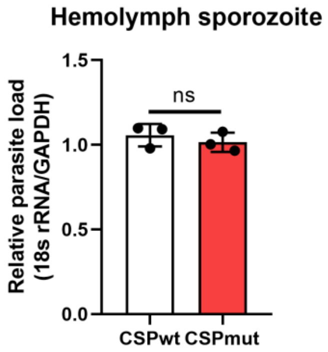

Supplementary Figure 2. The parasite load of CSP$_{wt}$ and CSP$_{mut}$ hemolymph sporozoites in HepG2-CD81 cells. 1×10^{5} HepG2-CD81 cells were infected with 5×10^{4} CSP$_{wt}$ or CSP$_{mut}$ hemolymph sporozoites from CSP$_{wt}$ or CSP$_{mut}$ parasite-infected mosquitoes (n = 30) for 42h. The parasite load was detected by qPCR. Three individual experiments have been performed.The data are presented as the means ± SD, and a two-tailed Student's t-test was used to calculate statistical significance. ns, no significance. Source data are provided as a Source Data file.

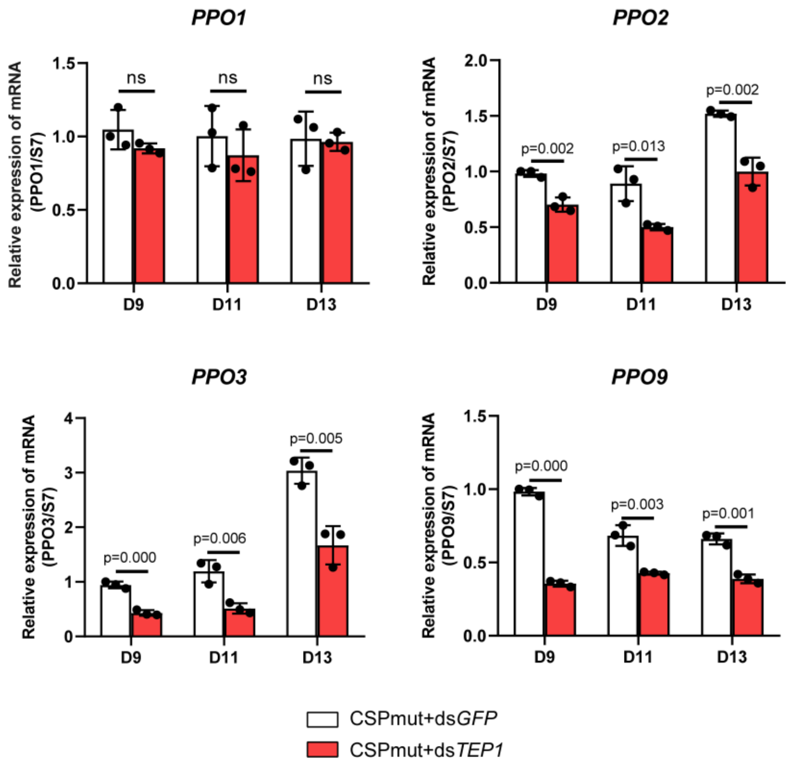

Supplementary Figure 3. The effect of *TEP1* knockdown on the expression of *PPO1*, *PPO2*, *PPO3* and *PPO9* in CSP$_{mut}$ parasite-infected mosquitoes. The mRNA levels of *PPO1*, *PPO2*, *PPO3* and *PPO9* in CSP$_{mut}$ parasite-infected mosquitoes (n=15) at the indicated time points were detected by

real-time PCR after *TEP1* was knockdown. Three individual experiments have been performed. The data are presented as the means ± SD, and a two-tailed Student’s t-test was used to calculate statistical significance. *p*-values are shown; ns, no significance. Source data are provided as a Source Data file.

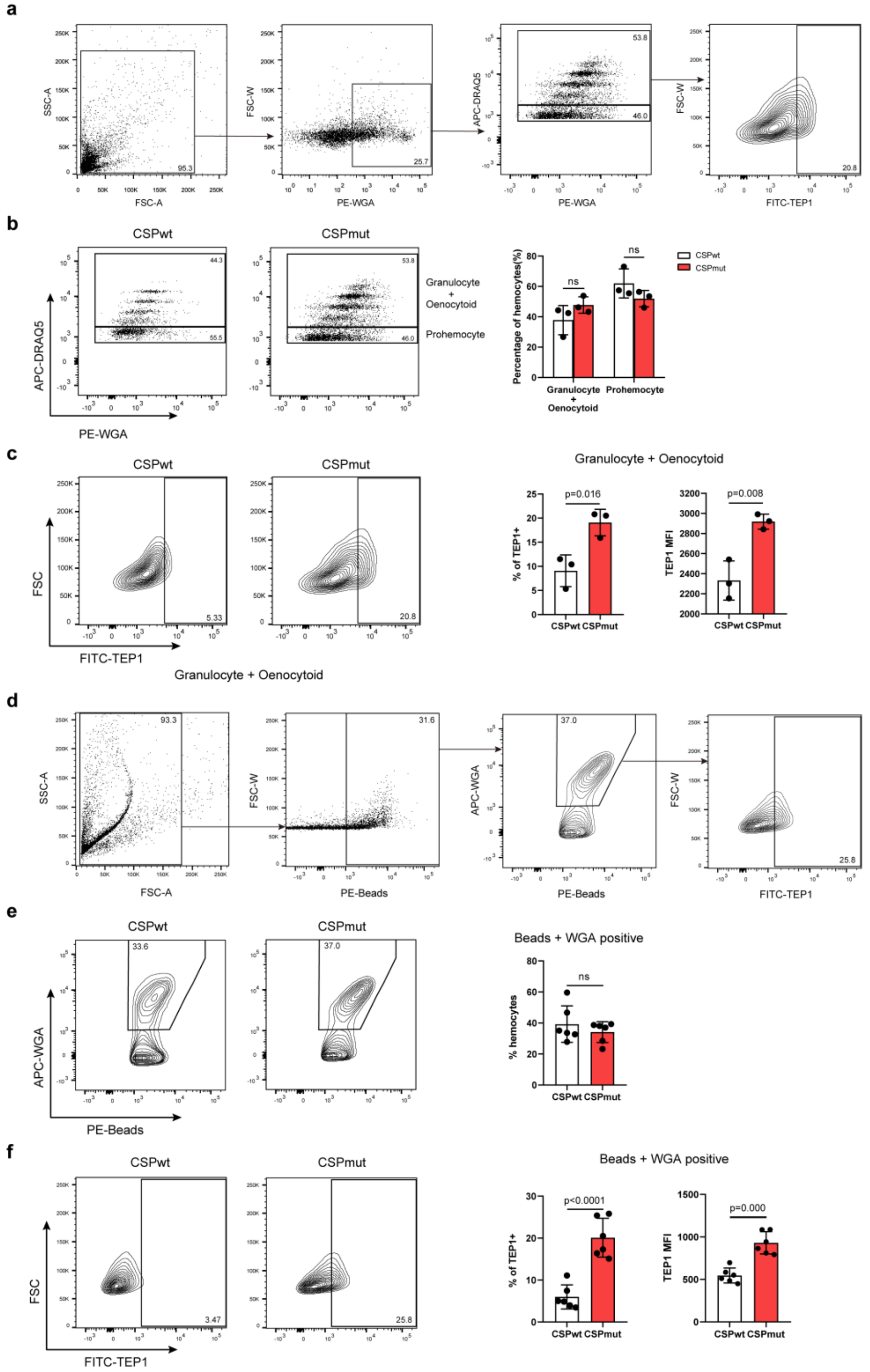

Supplementary Figure 4. The expression of TEP1 in hemocytes of CSP_{wt} and CSP_{mut} parasite-infected mosquitoes. Hemocytes collected from CSP_{wt} or CSP_{mut} parasites infected mosquitoes (n=60) at day 5 PI were stained with WGA, DRAQ5 and anti-TEP1 antibody conjugated with FITC, and then analyzed by FACS. **a** Gating strategy for flow cytometry with DRAQ5. **b** Representative FACS plot of three kinds of hemocytes from CSP_{wt} or CSP_{mut} parasite-infected mosquitoes (left), and the percentage of all three hemocytes from CSP_{mut} parasite-infected mosquitoes was compared to those from CSP_{wt} parasite-infected mosquitoes (right). **c** Representative FACS plot of TEP1 expression in granulocytes and oenocytoids from CSP_{wt} or CSP_{mut} parasite-infected mosquitoes (left), and the percentage of TEP1 positive granulocytes and oenocytoids, as well as the mean fluorescence intensity (MFI) of TEP1 were statistically analyzed (right). The experiment has been repeated for three times. **d** Gating strategy for flow cytometry with hemocytes phagocytosing of beads and expressing TEP1. **e** Representative FACS plot of bead-phagocytosed hemocytes from CSP_{wt} or CSP_{mut} parasite-infected mosquitoes (n=120) at day 5 PI (left), and the percentage of bead-phagocytosed hemocytes from CSP_{mut} parasite-infected mosquitoes was compared to those from CSP_{wt} parasite-infected mosquitoes (right). **f** Representative FACS plot of TEP1 expression in bead-phagocytosed hemocytes from CSP_{wt} or CSP_{mut} parasite-infected mosquitoes (left), and both the percentage and mean fluorescence intensity (MFI) of TEP1 in bead-phagocytosed hemocytes were statistically analyzed (right). The experiment has been repeated for three times. The data are presented as the means ± SD, and a two-tailed Student's t-test was used for comparison of two groups. *p*-values are shown; ns, no significance. Source data are provided as a Source Data file.

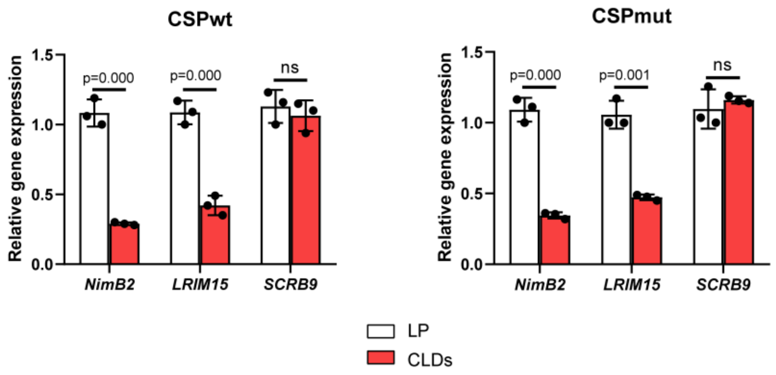

Supplementary Figure 5. The effect of clodronate liposomes on the depletion of mosquito hemocytes. Mosquitoes were injected with clodronate liposomes or the control liposomes (LP), and then were infected with CSP_{mut} parasite, 24 h later, hemocytes were perfused from the mosquitoes (n=15), and the expression of cell marker, *NimB2* (hemocytes), *LRIM15* (granulocytes) and *SCRB9* (oenocytoid) were determined by real-time PCR with *S7* as an internal control. The pooled data of three individual experiments was presented and analyzed. The data are presented as the means ± SD, and a two-tailed Student's t-test was used for comparison of two groups. *p*-values are shown; ns, no significance. Source data are provided as a Source Data file.

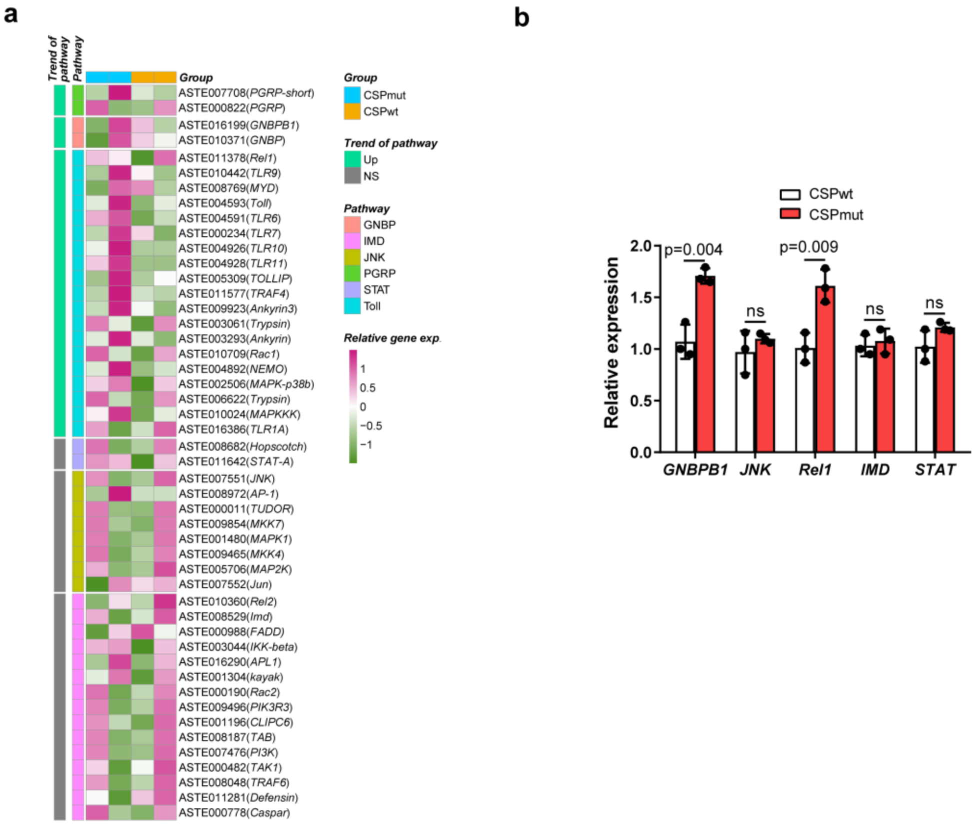

Supplementary Figure 6. The changes of immune-related pathways and genes in CSP_{wt} and CSP_{mut} parasites-infected mosquitoes.

a A heatmap comparing the differential expression of immune-related pathways and genes between CSP_{wt} and CSP_{mut} parasites-infected mosquitoes (n = 15) at day 7 PI; Up, upregulation, NS, no significance. Two biological repeats were performed. **b** Selected differentially expressed immune-related genes in both CSP_{wt}- and CSP_{mut}-infected mosquitoes (n = 15) at day 7 PI were validated using real-time PCR. This experiment was performed three times. The data are presented as the means ± SD, and a two-tailed Student's t-test was used for comparison of two groups. *p*-values are shown; ns, no significance. Source data are provided as a Source Data file.

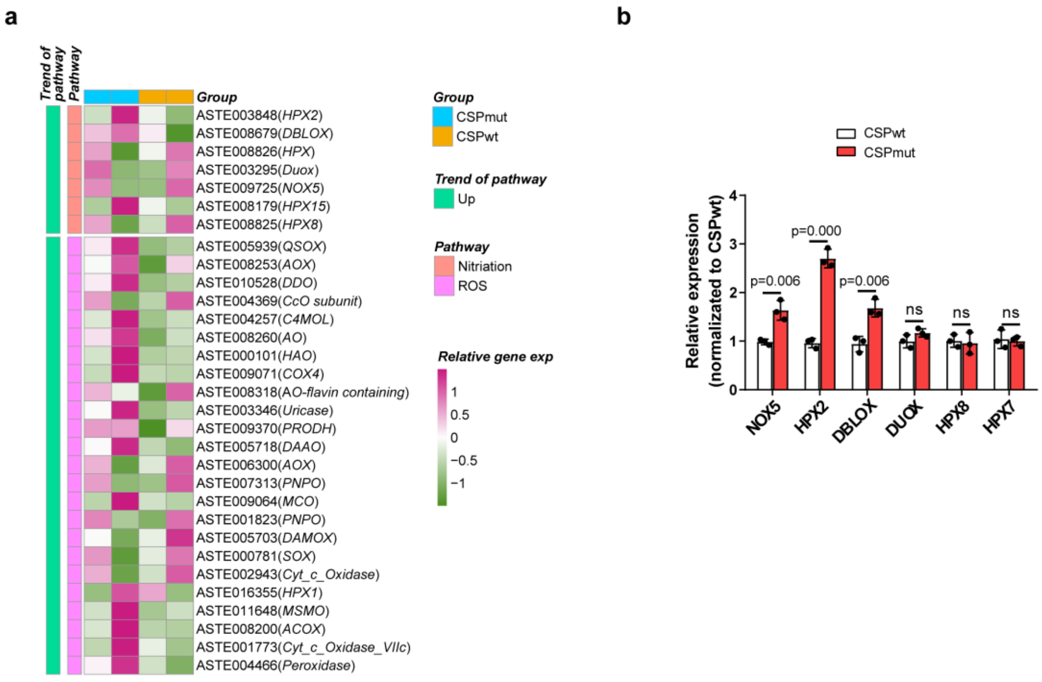

Supplementary Figure 7. The changes of Nitration and ROS pathways and genes in CSP_{wt} and CSP_{mut} parasites-infected mosquitoes.

a A heatmap of the differentially expressed reactive oxygen species, and nitration-related genes between WT and mutant parasite-infected mosquitoes (n=15) at day 7 PI, Up, upregulation, NS, no significance. Two biological repeats were performed. **b** The indicated differentially expressed genes in mosquitoes (n=15) infected with CSP_{wt} or CSP_{mut} at day 7 PI were validated by real-time PCR. The experiment was repeated three times. The data are presented as the means ± SD, and a two-tailed Student's t-test was used for comparison of two groups. *p*-values are shown; ns, no significance. Source data are provided as a Source Data file.

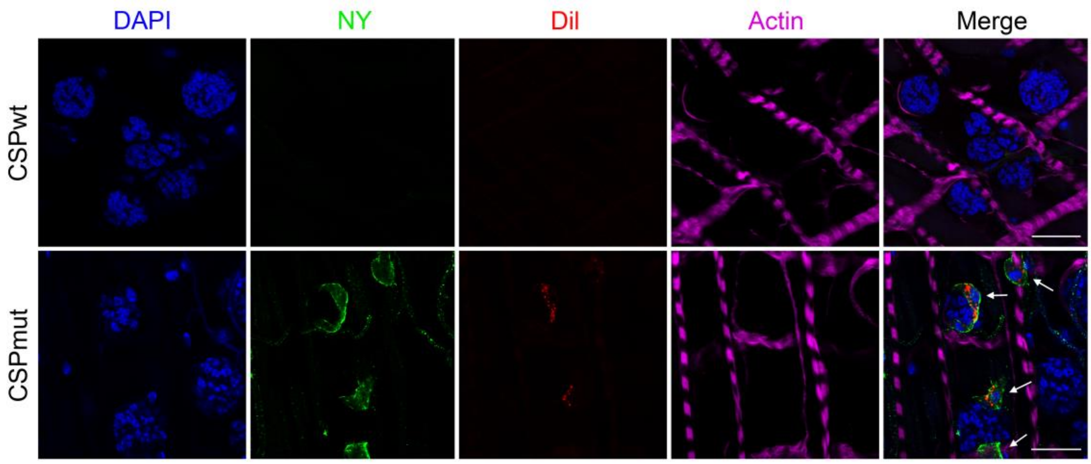

Supplementary Figure 8. Hemocytes attached to the mutant oocyst in mosquitoes. Mosquitoes were injected with Vybrant CM-Dil, and then infected with CSP_{wt} and CSP_{mut} parasites. At day 4 post infection, midguts were dissected from mosquitoes and fixed, and then stained with anti-nitrotyrosine, anti-Alexa Fluor 647 phalloidin and DAPI. The representative image of hemocytes attached to the mutant oocyst was presented. Experiment was performed twice. Sacle bar 50 µm.

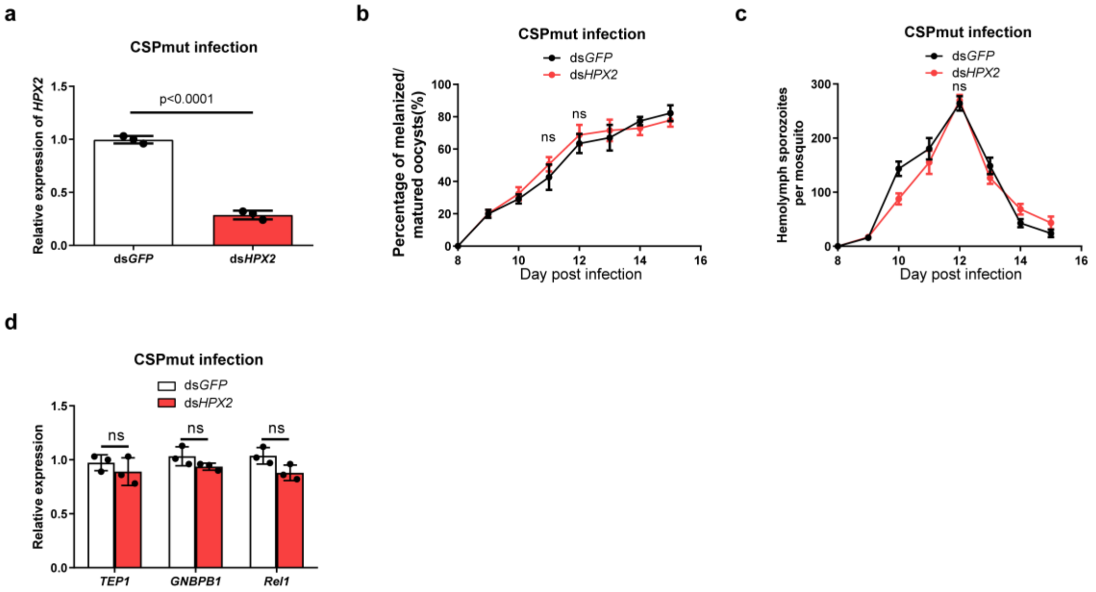

Supplementary Figure 9. The effect of *HPX2* knockdown on hemocyte nitration and CSP_{mut}

development in mosquitoes.

a The efficacy of RNA silencing of *HPX2* was determined by real-time PCR, and the relative expression of *HPX2* in mosquitoes (n=15) was expressed as the ratio of *HPX2* to the *S7* internal control. **b-d** The percentage of melanized mature mutant oocysts (**b**) in CSP_{mut} parasite-infected mosquitoes (n=24, pooled data), the number of hemolymph sporozoites (**c**) in CSP_{mut} parasite-infected mosquitoes (n=30, pooled data), the expression of *TEP1*, *GNBP-B1* and *Rel1* (**d**) in CSP_{mut} parasite-infected mosquitoes (n=15) at the indicated time points after *HPX2* knockdown. The experiments have been performed for two times. A two-tailed Student's t-test was used for comparison of two groups. The data are presented as the means ± SD. *p*-values are shown; ns, no significance. Source data are provided as a Source Data file.

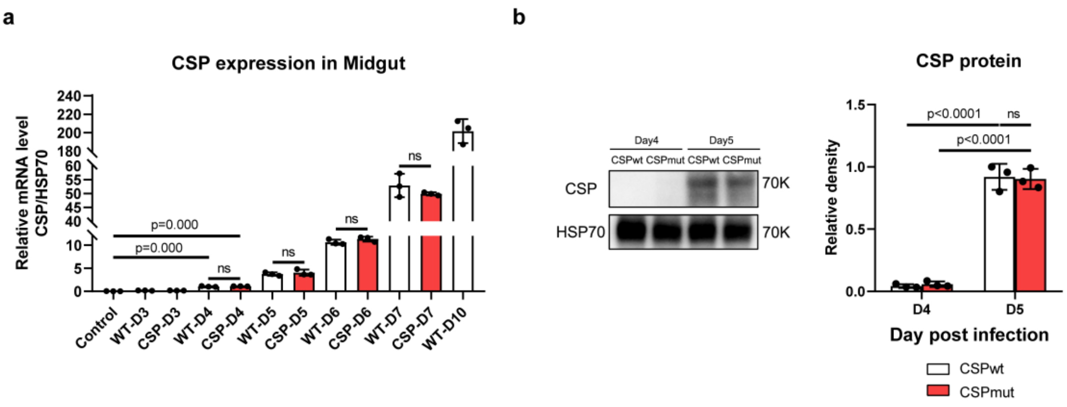

Supplementary Figure 10. The expression of CSP in the midgut of the CSP_{wt} and CSP_{mut} infected mosquitoes. a. The relative expression of *CSP* to *HSP70* in the oocysts of CSP_{wt} and CSP_{mut}-infected mosquitoes (n=15) at the indicated time points was determined by real-time PCR. **b.** The expression of CSP in the midgut of CSP_{wt} and CSP_{mut}-infected mosquitoes (n=40) at day 4 and 5 was detected by western blot, with HSP70 as the internal control. The experiment was repeated for three times. A two-tailed Student's t-test was used for comparison of two groups. The data are presented as the means ± SD. *p*-values are shown; ns, no significance. Source data are provided as a Source Data file.

Supplementary Movies

Supplementary Movie 1 (WT sporozoites)

Supplementary Movie 2 (Mut sporozoites)

Supplementary Tables

Supplementary Table 1

List of abbreviation and gene name in the heatmap comparing the differential expression of immune-related pathways genes and antioxidant genes

Pathway	Gene Number	Abbreviation of Gene Name	Gene Name	TPM (CSPmut1)	TPM (CSPmut2)	TPM (CSPwt1)	TPM (CSPwt2	Mean (CSPmut)	Mean (CSPwt)	Fold change （mut/ wt）
STAT	ASTE011642	STAT-A	signal transducer and transcription activator	34.10	33.22	26.02	32.92	33.66	29.47	**1.14**
	ASTE008682	Hopscotch	tyrosine-protein kinase hopscotch	24.83	5.86	10.01	23.94	15.34	16.97	0.90
JNK	ASTE007552	Jun	c-Jun N-terminal kinase	0.80	1.82	1.59	1.70	1.31	1.65	0.80
	ASTE005706	MAP2K	Mitogen-activated protein kinase kinase	49.88	21.87	26.38	61.15	35.87	43.77	0.82
	ASTE009465	MKK4	mitogen-activated protein kinase kinase 4	56.26	20.90	29.96	54.76	38.58	42.36	0.91
	ASTE001480	MAPK1	mitogen-activated protein kinase 1	62.63	18.52	25.93	64.13	40.57	45.03	0.90
	ASTE009854	MKK7	mitogen-activated protein kinase kinase 7	44.81	16.59	11.10	44.90	30.70	28.00	**1.10**
	ASTE000011	TUDOR	TUDOR-domain protein	42.05	17.83	17.31	42.81	29.94	30.06	1.00
	ASTE008972	AP-1	Activating protein-1	26.82	34.25	27.83	27.87	30.54	27.85	**1.10**
	ASTE007551	JNK	c-Jun N-terminal kinase	38.11	12.52	17.34	42.57	25.32	29.95	0.85
	ASTE000190	Rac2	Rac family small GTPase 2	22.73	3.85	9.83	21.44	13.29	15.64	0.85
	ASTE010709	Rac1	Rac family small GTPase 1	39.60	23.61	13.14	34.51	31.60	23.83	**1.33**
	ASTE010024	MAPKKK	Mitogen activated protein kinase-like protein	31.34	41.83	20.09	28.44	36.58	24.27	**1.51**
	ASTE002506	MAPK-p38b	p38b MAP kinase	56.19	61.14	40.06	56.66	58.67	48.36	**1.21**
**PGRP**	ASTE000822	PGRP	Peptidoglycan-recognition protein	381.73	80.26	102.83	330.32	230.99	216.58	**1.07**
	ASTE007708	PGRP (short)	Peptidoglycan recognition protein (short)	41.09	51.56	42.51	40.79	46.32	41.65	**1.11**
IMD	ASTE000778	Caspar	Caspar	125.28	58.57	46.11	111.51	91.93	78.81	**1.17**
	ASTE011281	Defensin	Defensin	5858.49	2619.29	6819.00	8384.31	4238.89	7601.66	0.56
	ASTE008048	TRAF	TNF-receptor-associated factor	46.39	16.77	26.92	52.45	31.58	39.69	0.80

	ASTE000482	TAK1	TGF-beta activated kinase 1	28.01	18.78	26.01	32.75	23.40	29.38	0.80
	ASTE007476	PI3K	phosphatidylinositol-4,5-bisphosphate 3-kinase	26.08	13.34	14.34	27.30	19.71	20.82	0.95
	ASTE008187	TAB	TAK1-associated binding protein	37.27	17.31	21.04	38.70	27.29	29.87	0.91
	ASTE001196	CLIPC6	serine protease persephone	65.16	55.00	49.47	67.29	60.08	58.38	**1.03**
	ASTE009496	PIK3R3	phosphoinositide-3-kinase, regulatory subunit	19.03	5.26	8.77	20.08	12.15	14.42	0.84
	ASTE001304	kayak (kay)	Transcription factor kayak	30.88	37.80	23.63	36.27	34.34	29.95	**1.15**
	ASTE016290	APL1	Anopheles Plasmodium-responsive leucine-rich repeat 1	105.02	210.61	92.04	169.23	157.82	130.63	**1.21**
	ASTE003044	IKK-beta	I-kappaB kinase beta	18.48	18.64	16.56	18.42	18.56	17.49	**1.06**
	ASTE000988	FADD	Fas-Associated Death Domain	8.90	12.73	14.66	11.75	10.81	13.20	0.82
	ASTE008529	Imd	Immune Deficiency (Imd)	30.56	13.02	22.20	35.90	21.79	29.05	0.75
	ASTE010360	Rel2	NF-kappaB Relish-like transcription factor	22.56	23.97	23.07	25.30	23.27	24.18	0.96
**GNBP**	ASTE010371	GNBP	beta-1,3-glucan-binding protein	5.36	15.34	12.03	10.38	10.35	11.21	0.92
	ASTE016199	GNBPB1	beta-1,3-glucan-binding protein B1	80.51	124.99	108.96	89.62	102.75	99.29	**1.03**
**Toll**	ASTE016386	TLR1A	TOLL-like receptor 1A	44.44	13.87	27.83	51.17	29.15	39.50	0.74
	ASTE006622	Trypsin	Trypsin serine proteases	18.84	14.62	12.67	17.96	16.73	15.32	**1.09**
	ASTE004892	NEMO	Nuclear factor kappa B essential modulator	72.01	112.85	58.23	70.56	92.43	64.39	**1.44**
	ASTE003293	Ankyrin	Ankyrin	4.22	6.99	4.21	3.09	5.60	3.65	**1.53**
	ASTE003061	Trypsin	Trypsin	14.47	11.82	9.04	14.21	13.15	11.63	**1.13**
	ASTE009923	Ankyrin3	Ankyrin	11.65	22.40	13.89	9.09	17.03	11.49	**1.48**
	ASTE011577	TRAF4	TNF Receptor-Associated Factor4	0.05	2.57	0.29	0.06	1.31	0.18	**7.34**
	ASTE005309	TOLLIP	Toll-interacting protein	44.90	65.08	45.79	51.74	54.99	48.76	**1.13**
	ASTE004928	TLR11	Toll-like receptor 11	2.41	3.22	1.44	1.41	2.81	1.43	**1.97**
	ASTE004926	TLR10	Toll-like receptor 10	0.63	1.14	0.47	0.47	0.88	0.47	**1.88**
	ASTE000234	TLR7	Toll-like receptor 7	0.57	1.57	1.00	0.29	1.07	0.64	**1.66**
	ASTE004591	TLR6	Toll-like receptor 6	0.48	0.62	0.07	0.25	0.55	0.16	**3.48**
	ASTE004593	Toll	Toll	0.12	0.34	0.03	0.12	0.23	0.07	**3.10**
	ASTE008769	MYD	Myeloid differentiation primary response protein MyD88	10.16	17.35	16.48	11.74	13.75	14.11	0.97
	ASTE010442	TLR9	Toll-like receptor 9	0.86	10.93	4.46	0.41	5.89	2.43	**2.42**
	ASTE011378	Rel1	NF-kappaB Relish-like transcription factor	96.00	89.54	55.64	107.53	92.77	81.58	**1.14**
**ROS**	ASTE004466	Peroxidase	Peroxidase	3.06	5.09	2.37	1.28	4.08	1.82	**2.24**
	ASTE001773	Cyt_c_Oxidase_VIIc	cytochrome c oxidase VIIc	1074.56	2125.26	1260.30	944.85	1599.91	1102.58	**1.45**
	ASTE008200	ACOX	Acyl-CoA oxidase	18.18	46.11	14.33	13.34	32.14	13.84	**2.32**
	ASTE011648	MSMO	Methylsterol monooxygenase	41.22	71.88	35.17	40.65	56.55	37.91	**1.49**
	ASTE016355	HPX1	Heme peroxidase 1	0.00	0.16	0.11	0.00	0.08	0.06	**1.39**
	ASTE002943	Cyt_c_Oxidase	Cytochrome C oxidase	115.52	63.78	89.32	131.63	89.65	110.48	0.81

	ASTE000781	FAD-linked SOX	Sulfhydryl oxidase	56.66	22.24	41.36	60.45	39.45	50.90	0.77
	ASTE005703	DAMOX	D-amino acid oxidase	18.85	12.34	17.97	26.93	15.60	22.45	0.69
	ASTE001823	PNPO	Pyridoxamine-phosphate oxidase	58.06	36.34	31.22	60.07	47.20	45.65	**1.03**
	ASTE009064	MCO	Multicopper oxidases	2.51	44.07	6.07	1.87	23.29	3.97	**5.87**
	ASTE007313	PNPO	pyridoxamine 5'-phosphate oxidase	41.17	26.01	27.38	45.57	33.59	36.48	0.92
	ASTE006300	AOX3	Acyl-coenzyme A oxidase	77.25	35.90	59.51	90.06	56.57	74.78	0.76
	ASTE005718	DAAO	D-amino-acid oxidase	124.04	212.12	90.11	63.47	168.08	76.79	**2.19**
	ASTE009370	PRODH	Proline dehydrogenase	184.33	182.29	60.86	159.75	183.31	110.30	**1.66**
	ASTE003346	Uricase	Uricase	61.49	146.49	13.21	31.75	103.99	22.48	**4.63**
	ASTE008318	Amine oxidase (flavin-containing)	Flavin-containing amine oxidase	11.69	11.11	10.00	12.21	11.40	11.10	**1.03**
	ASTE009071	COX4	Cytochrome c oxidase, subunit Vb/COX4	215.77	421.22	211.90	205.49	318.49	208.69	**1.53**
	ASTE000101	HAO	(S)-2-hydroxy-acid oxidase	14.84	69.81	5.90	9.12	42.32	7.51	**5.64**
	ASTE008260	AO	Amine oxidase	14.03	16.84	10.79	12.54	15.44	11.66	**1.32**
	ASTE004257	C4MOL	C-4 methylsterol oxidase	3.27	11.50	0.90	2.54	7.39	1.72	**4.30**
	ASTE004369	CcO subunit	Cytochrome c oxidase assembly protein subunit	55.97	42.89	46.95	59.08	49.43	53.02	0.93
	ASTE010528	DDO	D-aspartate oxidase	50.63	104.46	9.16	19.00	77.55	14.08	**5.51**
	ASTE008253	AOX	Acyl-coenzyme A oxidase	0.04	0.07	0.00	0.05	0.05	0.02	**2.31**
	ASTE005939	SOX	Sulfhydryl oxidase	75.96	100.78	55.55	60.81	88.37	58.18	**1.52**
**Nitriation**	ASTE008825	HPX8	Heme peroxidase 8	1647.32	407.10	958.89	1958.72	1027.21	1458.80	0.70
	ASTE008179	HPX15	Heme peroxidase 15	0.31	9.68	2.68	0.14	4.99	1.41	**3.55**
	ASTE009725	NOX5	NADH/NADPH oxidase and related proteins	23.77	7.97	8.20	26.24	15.87	17.22	0.92
	ASTE003295	Duox	Dual oxidase	88.93	12.45	18.55	82.26	50.69	50.41	**1.01**
	ASTE008826	HPX	Heme peroxidase	111.37	40.76	86.50	122.00	76.07	104.25	0.73
	ASTE008679	DBLOX	Double heme peroxidase	6.92	7.93	6.40	3.54	7.42	4.97	**1.49**
	ASTE003848	HPX2	Heme peroxidase 2	49.64	111.62	58.89	31.96	80.63	45.42	**1.78**

Note: The name of pathway in bold indicated that was upregulated for more than 1 fold in CSP_{mut}-infected mosquitoes, and the numbers of the upregulated fold of the related genes were also indicated as in bold.

Supplementary Table 2. The effect of knockdown of candidate genes on the melanization of mature mutant oocyst

Candidate Gene	*JNK*	*STAT*	*IMD*	*HPX2*	*DBLOX*	*DUOX*
Day10	31.4%	28.5%	0	54.5%	54.5%	73.4%
Day12	62.5%	66.7%	85.7%	66.6%	53.8%	76.4%
Day14	65.4%	85.7%	66.7%	65.7%	68.3%	84.6%

Note: "%" is the percentage of melanized mature mutant oocyst in the CSP_{mut} parasite-infected mosquitoes after the indicated genes was knockdown.

Supplementary Table 3.

List of primers used for Real time PCR analysis and for dsRNA synthesis

Primer Name	Primer Sequence (sequences are indicated from 5'to 3')
S7-QF	TCGGTTCCAAGGTGATCAAAGC
S7-QR	AGCGCGGTCTCTTCTGCTTGT
HSP70-QF	TGAAGCTGTATGCTCTCCAATTA
HSP70-QR	AGTTCATTCCTCCTGGCATTC
EGFP-T7F	TAATACGACTCACTATAGGTCAAGTTCAACGTGTCCGGCG
EGFP-T7R	TAATACGACTCACTATAGGAGGACCATTTGATCGCGCTT
TEP1- QF	AACTCGCAGGACATCAACATCACC
TEP1- QR	GGACGCTTCAGTGCCACCTTG
TEP1 -T7F	TAATACGACTCACTATAGGAACTCGCAGGACATCAACATCACC
TEP1 -T7R	TAATACGACTCACTATAGGGGACGCTTCAGTGCCACCTTG
Rel1- QF	GCTGTGCGAGAAGGTGGTGAAG
Rel1- QR	GGTGGCGTTCGGAAACTGATGG
Rel1- T7F	TAATACGACTCACTATAGGATCTGGTCGGTAAGGAGGGC
Rel1- T7R	TAATACGACTCACTATAGGGGTTCCGTAAAGCGTCCTCG
IMD-QF	GTGGTGGTGGTGGAGGAGGAG
IMD-QR	GGTGGTAGGCGTGTCGTTGAAC
IMD-T7F	TAATACGACTCACTATAGGGCCTCCTTCAGCTACACGAC
IMD-T7R	TAATACGACTCACTATAGGGTCGGTTCGCGGTTCAACT
JNK-QF	GGGCACGGTGTGGGAGTTTAAG
JNK-QR	GTGTACGCTGCGGAGTGAACG
JNK-T7F	TAATACGACTCACTATAGGCAAGCATCTCCACTCGGCTG
JNK-T7R	TAATACGACTCACTATAGGTTTCGCACGGTTGGCTGTAA
STAT-QF	GAGGTGACGGAGGTGTTCAATGC
STAT-QF	ATCAGGTTGCGGTTGTCGTTCTC
STAT-T7F	TAATACGACTCACTATAGGGCGGAGACGAACTTCACCATTAAGA
STAT-T7R	TAATACGACTCACTATAGGGGTTAATCTTCCACTGCGACAGATACTT
GNBPB1- QF	GTCGGCAATCGGCAGCACATAG
GNBPB1- QR	CTTCGGCAGCAACCAGATGGC
GNBPB1- T7F	TAATACGACTCACTATAGGACCAACAATCGGTCCAACTCGTTC
GNBPB1-T7R	TAATACGACTCACTATAGGGTTCTTCGTGAGCGTGGTCGTC
NOX5-QF	CAAGAAGCGGGAGCGTATGATGG
NOX5-QR	GTAAGCCAGACCGAGCGATTGAAG
NOX5-T7F	TAATACGACTCACTATAGGAAGGATGCCGAGGAAGGTCG
NOX5-T7R	TAATACGACTCACTATAGGCGTCGGTGGAATTTCGCTCG
HPX2-QF	ACCTACGCTGTCCTGTCTTATCCG
HPX2-QR	GCTGCACTGTACTCCGCCAAC
HPX2-T7F	TAATACGACTCACTATAGGCTCCGAGCGTCCTCCTTCTTC
HPX2-T7R	TAATACGACTCACTATAGGGGGATCGTCTTCGGGGACC
DBLOX-QF	GCGGTGGATACTTCAGCGGATAC

DBLOX-QR	CGGTGGAAGCATCGTGAGGAAC
DBLOX-T7F	TAATACGACTCACTATAGGGCGAGGTGACGAACCATCTG
DBLOX-T7R	TAATACGACTCACTATAGGAGCTGCTCGCTGCATATCAC
DUOX-QF	CGAGATAGTCATGGCGTCCGAATC
DUOX-QR	GCACGGTGGAACGGTATGTAGC
DUOX-T7F	TAATACGACTCACTATAGGGGACGGTGAGGTAATGACGG
DUOX-T7R	TAATACGACTCACTATAGGAGTCACGCTTGTGGTTCGAG
PPO1-QF	GGTCAACTTCCTCACGCCCAAC
PPO1-QR	CCTGCCAGCATATAGACGGATAAGC
PPO2-QF	AGTCAGACCAACCGTGCCTACC
PPO2-QR	GCCACCGTTCGAGATCGTTCAC
PPO3-QF	GCAGGCGGAGAACAGAATGACC
PPO3-QR	CCAGATGCCAGTGCCAGTGATG
PPO9-QF	GACGCTACGGTACAGATAACGAGTG
PPO9-QR	CGTGCTGGATGTGGGTAAAGGTC
NimB2-QF	TACCGCTGGGCAATGGATCAAATC
NimB2-QR	CTCGCATACGGGCTCACACTTG
LRIM15-QF	GTCCTAACGCTGAACCACAATTTGC
LRIM15-QR	GCCACGATTGATGCCGATCCTC
SCRB9-QF	GACTAGCGATGGCGAGCATTATGG
SCRB9-QR	CTTGAGCAGCGGATCTTCGTAGC
CSP-QF	ACAACAGCCACCACAACAAC
CSP-QR	CACTACATTGAGACCATTCCTCTG
18s rRNA-QF	GCAGCAACGGCTCCATGACTC
18s rRNA-QR	CTCCTCCTGGTAGATGTGGTCCTC
Probe for 18s rRNA	FAM-AACCTTCCCAAAAAT-MGB
human GAPDH-QF	GGACCTGACCTGCCGTCTAG
human GAPDH-QR	CCTGCTTCACCACCTTCTTGA
Probe for human GAPDH	FAM-AACCTGCCAAATATGATGAC-MGB

Supplementary Table 4

Polypeptide and antigenic sequence designed for the preparation of polyclonal antibodies

Sequence Name	Sequence
anti-CSP-N-terminal	LNSKNGKIYNRN
anti-CSP-C-terminal	KNVNKQPENLTLE
anti-CSP-repeat	QGPGAPQGPGAP-QGPGAPQGPGAP- QGPGAPQGPGAP
anti-TEP1	LAEKISPSRNDYTITLKYKRSVRNFYINSQDINITSYEDIPEDTRALEVNVGGIGFGLL QVIYQYSLNLVNFAHRFKLDLERQSTGSEYELRMRVCANFIPKMTDSRS
anti-HSP70	MANAKASKPNLPESNIAIGIDLGTTYSCVGVWRNENVDIIANDQGNRTTPSYVA FTDTERLIGDAAKNQVARNPENTVFDAKRLIGRKFTESSVQSDMKHWPFTVKSG IEEKPMIEVVYQGEKKLFHPEEISSMVLQKMKENAEAFLGKSIKNAVITVPAYFND SQRQATKDAGTIAGLNVMRIINEPTAAAIAYGLHKKGKGEKNILIFDLGGGTFDVS LLTIEDGIFEVKATAGDTHLGGEDFDNRLVNFCVEDFKRKNRGKDLSKNSRALRRL RTQCERAKRTLSSSTQATIEIDSLFEGIDYSVTVSRARFEELCIDYFRDTLIPVEKVLK DAMMDKKSVHEVVLVGGSTRIPKIQTLIKEFFNGKEACRSINPDEAVAYGAAVQ AAILSGDQSNAVQDLLLLDVCSLSLGLETAGGVMTKLIERNTTIPAKKSQIFTTYAD NQPGVLIQVYEGERALTKDNNLLGKFHLDGIPPAPRKVPQIEVTFDIDANGILNVT AVEKSTGKQNHITITNDKGRLSPEEIDRMVNDAEKYKAEDEENKKRIEARNSLEN YCYGVKSSLEDQKIKEKLQPNEVETCMKSVTSILEWLEKNQLAGKDEYEAKQKEA EAVCSPIMSKIYQDAGAAAGGMPGGMPGGMPGGMPGGMPGGMNFPGGM PGGMGAPAGAPAGSGPTVEEVD
anti-S7	MVFGSKVIKAGNGEPDAFETQIGQAILELEMNSDLKPQLRDLYITRAREVEFNNK KAIIIYVPVPKQKAFQKVQTRLVRELEKKFSGKHVVFIAERRILPKPMRGRRDPNK QKRPRSRTLTAVYDAILEDLVFPAEVVGKRIRVKLDGSQLIKVHLDKNQQTTIEHK VDTFASVYKKLTGRDVTFEFPENYL

Supplementary methods

The expression difference of TEP1 in hemocytes determined by flow cytometry

According to the methods previously described[1,2], hemolymph was perfused from CSP_{wt} or CSP_{mut}-infected mosquito (n=60) at 5 day post-feeding. Collected hemolymph was washed with 1× PBS to a final volume of 1 mL, then centrifuged at 2000g for 5 min to pellet cells. After removing the supernatant, cells were washed two times in 1× PBS with an additional centrifugation step of 5 min at 2000g between washing steps. For DRAQ5 staining, cells were incubated with WGA Alexa Fluor 555 (1:5000, invitrogen) and DRAQ5 (1:1000, Thermo Fisher Scientific) for 60 min in the dark at room temperature. For phagocytic assay of fluorescent FluoSpheres, CSP_{wt} or CSP_{mut}-infected mosquito (n=60) at 4 day PI were injected with red fluorescent FluoSpheres (1 μm; Molecular Probes) at a final concentration of 2% (vol/vol). The next day, hemocytes were collected by perfusion and incubated with WGA Alexa Fluor 647 (1:5000, invitrogen). After incubation, cells

were washed two times in 1× PBS to remove excess stain with a centrifugation step of 5 min at 2000g. The centrifuged cells were incubated with PBT (1× PBS containing 1%BSA and 0.1% Triton X-100) for 30 min at room temperature. PBT was removed and cells were incubated for 1 h in the dark with anti-TEP1 Polyclonal antibody (1mg/ml, 1:1000 dilution) coupled by FITC fast Conjugation Kit (abcam). And then, cells were run on a BD FACSCanto II cytometer (BD Biosciences), according to the previous flow cytometry condition for establishment of threshold values for gating[1,2]. Smaller cells or larger than single cells were ruled out. Following preliminary gating for cell size, the second gating was based on WGA signals, cell populations were distinguished by WGA and DRAQ5 or fluorescent FluoSpheres signals. Next, the expression difference of TEP1 in cell populations was discriminated by coupling FITC signal.

RNA-Seq and differential gene expression analysis

On day 7 after blood feeding, TRIzol™ Reagent (Thermo Fisher Scientific) was used to extract total RNA from a pool of 15 whole mosquitoes infected with CSP_{wt} or CSP_{mut} parasites. High-quality RNA samples were subsequently submitted to Sangon Biotech Co., Ltd. (Shanghai, China) for RNA-sequencing and data analysis. Clean reads were mapped to the reference genome downloaded from VectorBase (Anopheles_stephensi_AsteI2 genome)[3], using HISAT2 v2.0 (http://daehwankimlab.github.io/hisat2/) with default parameters. Transcripts per million (TPM) analysis eliminated the influence of gene lengths and sequencing discrepancies, to enable direct comparison of gene expression between samples. DESeq2 v1.12.4 was used to determine differentially expressed genes (DEGs) between the two samples. Genes were considered to be significantly differentially expressed if the q-value < 0.001 and $|$ fold change $| > 1.2$. Gene expression data are deposited in NCBI's Gene Expression Omnibus and is accessible through GEO Series accession number GSE176061 (https://www.ncbi.nlm.nih.gov/geo/query/acc.cgi?acc=GSE176061). DEGs were validated using qPCR as described above, and the primer sequences are shown in Supplementary Table 2. Differently expressed genes in supplementary Figure.6a and 7a were listed in Supplementary Table 4.

The parasite load of CSP_{wt} and CSP_{mut} hemolymph sporozoites in HepG2-CD81 cells.

At 12 day after infection with CSP_{wt} or CSP_{mut}-infected mosquito, hemolymph was perfused from the mosquitoes (n=60), as described above. Hemolymph sporozoites were collected with centrifugation at 12,000g for 10 min and resuspended in RPMI 1640 medium containing 2.5 μg/mL amphotericin B (Sangon Biotech, Shanghai, China),100 units/ml penicillin, and 100 μg/ml streptomycin (Beyotime, Beijing, China). Sporozoites were counted and incubated with HepG2-CD81 cells (a gift from Prof. Jing Yuan, Xiamen Univeristy) at a ratio of 1:2. After infection with three hours, the supernatant was removed and added fresh culture medium with the three antibiotics as described above. For parasite burden analysis, HepG2-CD81 cells were collected at 42h after infection with CSP_{wt} or CSP_{mut} sporozoites and lysed with 1 mL Trizol (Invitrogen) according to the instructions. 2 μg RNA was used for first-strand cDNA synthesis with the PrimeScript™ RT reagent Kit with gDNA Eraser (Perfect Real Time) (Takara Bio Inc., Japan). The parasite load in the HepG2-CD81 cells was evaluated by Taqman PCR with primers and probes for *18S rRNA* and *GAPDH* (Supplementary Table 3) following the manufacturer's instructions for Premix EX Taq™ (Probe qPCR) (TAKARA). The relative parasite load was expressed as the ratio of *18S rRNA* to *GAPDH*.

The motility test of hemolymph sporozoite

To test the motility of hemolymph sporozoite, hemolymph was perfused from female mosquitoes from CSP_{wt} or CSP_{mut}-infected mosquitoes (12 days post-feeding), hemolymph sporozoite were collected with centrifugation at 12000g for 3 min and resuspended in PBS. Sporozoites were incubated for 30 min at 37°C with anti-CSP-C terminal antibody diluted (1:100) in PBS. Then, samples were washed 2 times with PBS and incubated for 30 min at 37°C with Alexa 488-conjugated secondary antibodies (Beyotime Biotech, Nantong, China) diluted (1:100) in PBS. Sporozoites were collected with centrifugation of 3 min at 12,000g and resuspended in PBS. The sporozoites were placed in hemocytometer and the motility of sporozoites was observed with fluorescence microscope (IX71, Olympus, Japan).

References

1. Kwon, H. & Smith, R. C. Chemical depletion of phagocytic immune cells in Anopheles gambiae reveals dual roles of mosquito hemocytes in anti-Plasmodium immunity. *Proc Natl Acad Sci U S A*.

116, 14119-14128 (2019).

2. Kwon, H., Mohammed, M., Franzen, O., Ankarklev, J. & Smith, R. C. Single-cell analysis of mosquito hemocytes identifies signatures of immune cell subtypes and cell differentiation. *ELife*. **10**, (2021).
3. Giraldo-Calderon, G. I. et al. VectorBase: an updated bioinformatics resource for invertebrate vectors and other organisms related with human diseases. *Nucleic Acids Res.* **43**, D707-D713 (2015).

Full blots and gels of results are shown

Fig.2f

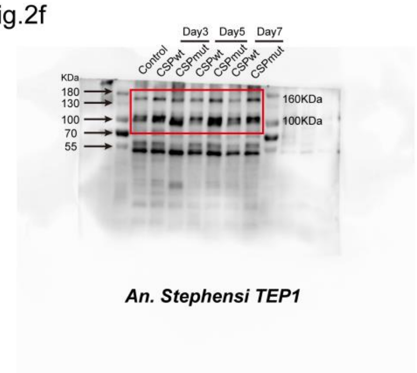

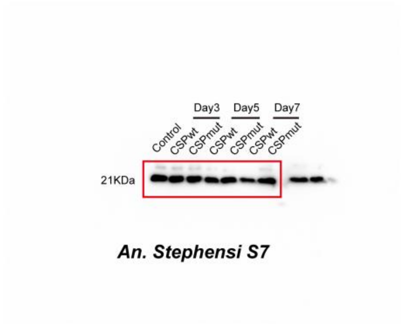

Fig.5a

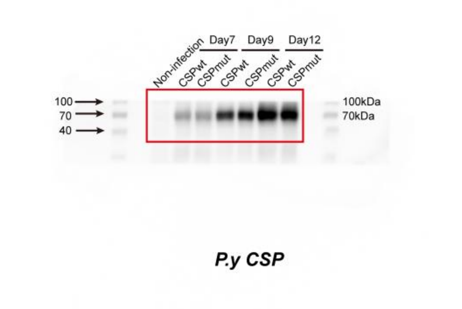

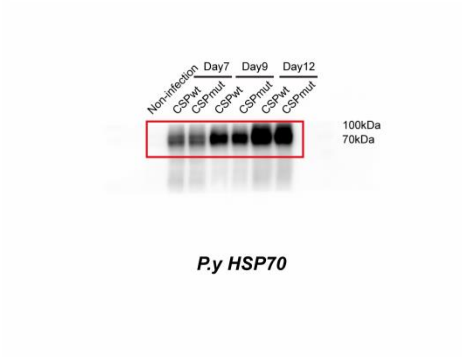

Fig.S1b

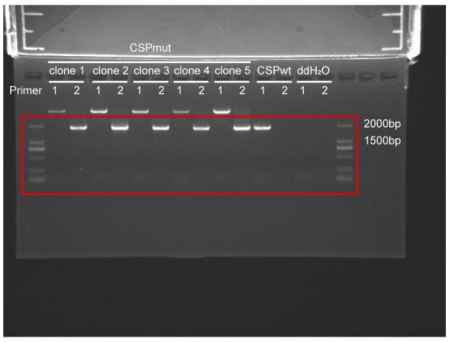

Fig.S10b

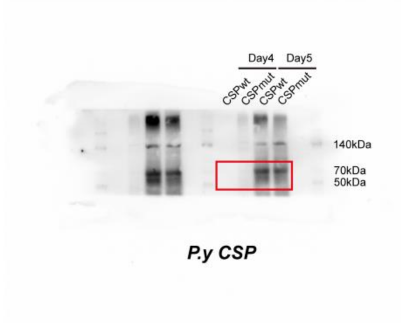

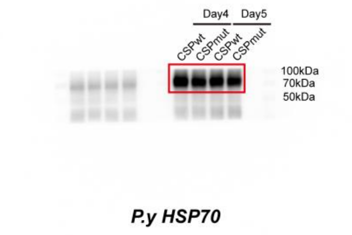

Supplement: Supplementary file 1 — Supplementary Information [file 41467_2022_30988_MOESM1_ESM.pdf]
