# Supplementary material for: Malaria oocysts require circumsporozoite protein to evade mosquito immunity
Source: Nat Commun. 2022 Jun 9;13:3208. doi: 10.1038/s41467-022-30988-z (PMC9184642; doi:10.1038/s41467-022-30988-z)
Supplement: Supplementary file 3 — Description of Additional Supplementary Files [file 41467_2022_30988_MOESM3_ESM.pdf]

Description of Additional Supplementary Files

File Name: Supplementary Movie 1
Description: The motility of hemolymph sporozoites from CSP_{wt} parasite-infected mosquitoes.

File Name: Supplementary Movie 2
Description: The motility of hemolymph sporozoites from CSP_{mut} parasite-infected mosquitoes.
