# Supplementary figures and images for: Malaria oocysts require circumsporozoite protein to evade mosquito immunity
Source: Nat Commun. 2022 Jun 9;13:3208. doi: 10.1038/s41467-022-30988-z (PMC9184642; doi:10.1038/s41467-022-30988-z)

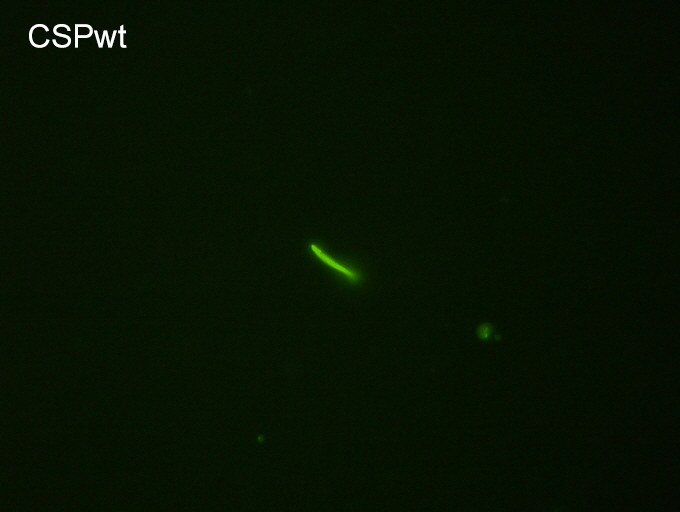

Supplement: Supplementary file 4 — Supplementary Movie 1 [file 41467_2022_30988_MOESM4_ESM.gif]

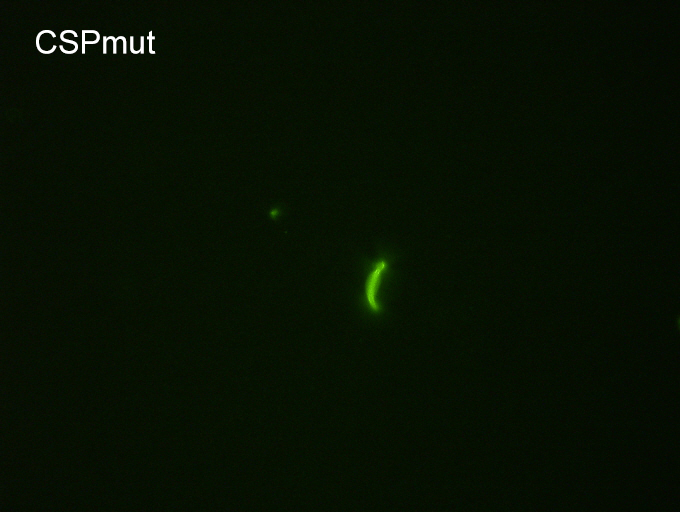

Supplement: Supplementary file 5 — Supplementary Movie 2 [file 41467_2022_30988_MOESM5_ESM.gif]
