# Supplementary material for: Malaria oocysts require circumsporozoite protein to evade mosquito immunity
Source: Nat Commun. 2022 Jun 9;13:3208. doi: 10.1038/s41467-022-30988-z (PMC9184642; doi:10.1038/s41467-022-30988-z)
Supplement: Supplementary file 6 — Reporting Summary [file 41467_2022_30988_MOESM6_ESM.pdf]

nature portfolio

Corresponding author(s): Wenyue, Xu; Jian Zhang

Last updated by author(s): May 4, 2022

Reporting Summary

Nature Portfolio wishes to improve the reproducibility of the work that we publish. This form provides structure for consistency and transparency in reporting. For further information on Nature Portfolio policies, see our Editorial Policies and the Editorial Policy Checklist.

Statistics

For all statistical analyses, confirm that the following items are present in the figure legend, table legend, main text, or Methods section.

n/a	Confirmed	
☐	☒	The exact sample size (*n*) for each experimental group/condition, given as a discrete number and unit of measurement
☐	☒	A statement on whether measurements were taken from distinct samples or whether the same sample was measured repeatedly
☐	☒	The statistical test(s) used AND whether they are one- or two-sided<br>*Only common tests should be described solely by name; describe more complex techniques in the Methods section.*
☒	☐	A description of all covariates tested
☐	☒	A description of any assumptions or corrections, such as tests of normality and adjustment for multiple comparisons
☐	☒	A full description of the statistical parameters including central tendency (e.g. means) or other basic estimates (e.g. regression coefficient) AND variation (e.g. standard deviation) or associated estimates of uncertainty (e.g. confidence intervals)
☐	☒	For null hypothesis testing, the test statistic (e.g. *F*, *t*, *r*) with confidence intervals, effect sizes, degrees of freedom and *P* value noted<br>*Give P values as exact values whenever suitable.*
☒	☐	For Bayesian analysis, information on the choice of priors and Markov chain Monte Carlo settings
☒	☐	For hierarchical and complex designs, identification of the appropriate level for tests and full reporting of outcomes
☒	☐	Estimates of effect sizes (e.g. Cohen's *d*, Pearson's *r*), indicating how they were calculated

Our web collection on statistics for biologists contains articles on many of the points above.

Software and code

Policy information about availability of computer code

Data collection	no software was used.
Data analysis	Clean reads of RNA-seq were mapped to the reference genome downloaded from VectorBase (Anopheles_stephensi_AsteI2 genome)3, using HISAT2 v2.0 (http://daehwankimlab.github.io/hisat2/) with default parameters. DESeq2 v1.12.4 was used to determine differentially expressed genes (DEGs) between the two samples. Heatmap showing differences in gene expression was created by Package pHeatmap v1.0.12 in R (4.0.2, https://www.R-project.org/).

For manuscripts utilizing custom algorithms or software that are central to the research but not yet described in published literature, software must be made available to editors and reviewers. We strongly encourage code deposition in a community repository (e.g. GitHub). See the Nature Portfolio guidelines for submitting code & software for further information.

Data

Policy information about availability of data

All manuscripts must include a data availability statement. This statement should provide the following information, where applicable:

- Accession codes, unique identifiers, or web links for publicly available datasets
- A description of any restrictions on data availability
- For clinical datasets or third party data, please ensure that the statement adheres to our policy

Data are publicly available from the Gene Expression Omnibus (GEO) (accession code GSE176061).

Field-specific reporting

Please select the one below that is the best fit for your research. If you are not sure, read the appropriate sections before making your selection.

☒ Life sciences ☐ Behavioural & social sciences ☐ Ecological, evolutionary & environmental sciences

For a reference copy of the document with all sections, see nature.com/documents/nr-reporting-summary-flat.pdf

Life sciences study design

All studies must disclose on these points even when the disclosure is negative.

Sample size	No statistical methods were used to predetermine sample sizes. Samples sizes were determined based on previous relevant studies (Ramirez, J., Nat Commun, 2015; Kumar, S.,Science, 2010) about observing the development of the parasites in mice and mosquito, quantitative real-time PCR analysis, gene silencing, Immunofluorescence analysis, Western blot analysis and flow cytometry. Samples sizes adopted in this study were sufficient for detecting robust effects.
Data exclusions	No samples or animals were excluded from the analysis.
Replication	All observing the development of the parasites in mice and mosquito, quantitative real-time PCR analysis gene silencing, Immunofluorescence analysis, Western blot analysis, flow cytometry and in vitro experiments were performed over at least 2-3 independent replicates. The study effects were all successfully replicated in the experiments.
Randomization	For all experiments, samples were randomized where appropriate for data collection and analysis.
Blinding	In the studies, investigators were blinded to groups allocation during data collection and analysis.

Reporting for specific materials, systems and methods

We require information from authors about some types of materials, experimental systems and methods used in many studies. Here, indicate whether each material, system or method listed is relevant to your study. If you are not sure if a list item applies to your research, read the appropriate section before selecting a response.

Materials & experimental systems

n/a	Involved in the study	
☐	☒	Antibodies
☐	☒	Eukaryotic cell lines
☒	☐	Palaeontology and archaeology
☐	☒	Animals and other organisms
☒	☐	Human research participants
☒	☐	Clinical data
☒	☐	Dual use research of concern

Methods

n/a	Involved in the study	
☒	☐	ChIP-seq
☐	☒	Flow cytometry
☒	☐	MRI-based neuroimaging

Antibodies

Antibodies used	The immunostaining, western blot and flow cytometry were performed with the following antibodies: Anti-CSP-N, anti-CSP-C, anti-CSP-repeat-region, and anti-TEP1 were synthesized by GeneCreate Biotech (G1061, G1062, G1080, G1052, rabbit polyclonal antibody ,Wuhan, China), anti-P. yoelii HSP70 and anti- A. stephensi S7 rabbit polyclonal antibody (pCzn1 and pet B2M expression vector, E. coli Arctic-ExpressTM), Cy3-labeled and Alexa 488-conjugated goat anti-rabbit IgG, Alexa 647-conjugated goat anti-mouse IgG (Beyotime Biotech, A0516,Lot#062521211011, A0423, Lot#031221210921, A0473, Lot#041221210915), anti-nitrotyrosine mouse monoclonal antibody (Abcam, ab7048,Lot#GR3287297-1), horseradish peroxidase labeled goat anti-rabbit IgG (Zhongshan Golden Bridge Biotechnology, ZB-2301, Lot#205001023).
Validation	The application and specificity of all the antibodies are validated by the companies. The primary antibodies: Anti-CSP-N, anti-CSP-C, anti-CSP-repeat-region, anti-TEP1 anti-P. yoelii HSP70 and anti- A. stephensi S7 were rabbit polyclonal antibodies that were validated by Western Blot and ELISA, and were diluted in PBS with 1:200 for the immunostaining and 1:1000 for the flow cytometry with 1:5000 for western blot. anti-nitrotyrosine cat NO. ab7048,is a mouse monoclonal antibody that reacts with nitrotyrosine, and is tested for use in Immunohistochemistry. Please visit the company's website for details. https://www.abcam. cn/nitro-tyrosine-antibody-hm11-ab7048.html.

Eukaryotic cell lines

Policy information about cell lines

Cell line source(s)	HepG2 cell line was come from ATCC（NO: HB-8065），and HepG2-CD81 was constructed from the HepG2 by Prof. Jingyuan, Xiamen University of China.
Authentication	The cell lines were authenticated via STR profiling.
Mycoplasma contamination	HepG2-CD81 cells were tested negative for Mycoplasma contamination.
Commonly misidentified lines (See ICLAC register)	no commonly misidentified cell lines were used in the study.

Animals and other organisms

Policy information about studies involving animals; ARRIVE guidelines recommended for reporting animal research

Laboratory animals	Kunming mice (Female, 6-8 weeks old) were provided by the Laboratory Animal Center of the Army medical University (Chongqing, China); BALB/c and C57BL/6J mice (Female, 6-8 weeks old) purchased from Beijing Animal Institute (Beijing, China). The mice were kept in a temperature- and humidity-controlled room on a cycle of 12 h light/dark (lights off at 19:00).<br>A. stephensi from our laboratory was maintained at 27 °C at a relative humidity of 70 %–80 %, and fed with a 10 % sugar solution containing 0.5 % para-aminobenzoic acid (PABA). The room was on a cycle of 12 h light/dark (lights off at 18:00).<br>Both P. yoelii 265BY and P. yoelii 265BY-RFP, which was constructed using a single cross-mediated insertion of RFP in the SSU rRNA gene of the P. yoelii 265BY parasite45, were maintained by alternate passaging between Kunming mice.
Wild animals	The study did not involve wild animals.
Field-collected samples	The study did not involve samples collected from the field.
Ethics oversight	All animal protocols were reviewed and approved by the Animal Ethics Committee of the Army Medical University Institute of Medical Research (AMUWEC20181777).

Note that full information on the approval of the study protocol must also be provided in the manuscript.

Flow Cytometry

Plots

Confirm that:

- [x] The axis labels state the marker and fluorochrome used (e.g. CD4-FITC).
- [x] The axis scales are clearly visible. Include numbers along axes only for bottom left plot of group (a 'group' is an analysis of identical markers).
- [x] All plots are contour plots with outliers or pseudocolor plots.
- [x] A numerical value for number of cells or percentage (with statistics) is provided.

Methodology

Sample preparation	For DRAQ5 staining, hemolymph was perfused from female mosquitoes (n=60) from CSPwt or CSPmut-infected mosquito at 5 day post-feeding. Collected hemolymph was washed with 1X PBS to a final volume of 1 mL, then centrifuged at 2000g for 5 min to pellet cells. For phagocytic assay of fluorescent FluoSpheres, CSPwt or CSPmut-infected mosquito (n=60) at 4 day PI were injected with red fluorescent FluoSpheres (1 µm; Molecular Probes) at a final concentration of 2% (vol/vol). The next day, hemocytes were collected by perfusion.
Instrument	BD FACSCanto II
Software	FlowJo_v10.8.1
Cell population abundance	Cells were run on a BD FACSCanto cytometer (BD Biosciences), according to the previous flow cytometry condition for establishment of threshold values for gating (Kwon and Smith, 2019, 2021). Smaller cells or larger than single cells were ruled out (Threshold =20000). Average ratio and number of Hemocytes (WGA positive) in infected mosquitoes were 25% and 2662,respectively.
Gating strategy	Following preliminary gating for cell size, for DRAQ5 staining, the second gating was based on WGA signals, cell populations were distinguished by WGA and DRAQ5 signals; for phagocytic assay of fluorescent FluoSpheres, the second gating was based on fluorescent FluoSpheres signals, cell populations were distinguished by fluorescent FluoSpheres and WGA signals. Next, the expression difference of TEP1 in cell populations was discriminated by coupling FITC signal.

- [x] Tick this box to confirm that a figure exemplifying the gating strategy is provided in the Supplementary Information.
